# Non‐Native Plants Alter Bird‐Plant Frugivory Network Structure in a Human‐Modified Tropical Landscape

**DOI:** 10.1002/ece3.72620

**Published:** 2025-12-09

**Authors:** Adam Fell, Carolina Bello, A. Bradley Duthie, Marta Vargas, Alastair Skeffington, Kristin Saltonstall, Daisy H. Dent

**Affiliations:** ^1^ Biological and Environmental Sciences University of Stirling Stirling UK; ^2^ Department of Environmental Systems Science Eth Zurich Switzerland; ^3^ Smithsonian Tropical Research Institute Balboa Panama; ^4^ Max Planck Institute of Animal Science Konstanz Germany

**Keywords:** frugivorous birds, frugivory network, metabarcoding, non‐native plants, seed dispersal, tropical landscape

## Abstract

Frugivory interactions are key plant–animal mutualisms that facilitate seed dispersal and promote ecosystem resilience. However, these interaction networks are increasingly altered by the widespread introduction of non‐native plants through human activities. The integration of such species into frugivory networks—and their consequences for network stability—remains poorly understood. Here, we examined the role of non‐native plants in shaping frugivory network structure and *robustness* in a human‐modified tropical landscape. Using DNA metabarcoding of faecal samples from 21 frugivorous bird species in Gamboa, Panama, we identified consumed plant species and quantified the contribution of non‐natives to avian diets. Non‐native plants significantly altered network structure, reducing *nestedness* while increasing *connectance* and *modularity* compared to native‐only networks. Extinction simulations revealed that non‐native plants, despite comprising only 28% of plant species, triggered disproportionately higher secondary bird extinctions. Yet, these species showed lower persistence during sequential bird removals, creating a paradox: they act as crucial connectors within the network while simultaneously undermining its stability. Notably, three non‐native species served as key connector species linking network modules. The high connectivity of certain non‐native connector species is particularly concerning given their documented invasive potential in other regions. We anticipate that these findings will inform conservation strategies in human‐modified landscapes, particularly regarding the monitoring and management of highly connected non‐native plants that may both compromise ecosystem stability and facilitate biological invasions. While non‐native plants may provide temporary alternative food resources for adaptable frugivorous birds navigating increasingly human‐modified environments, they simultaneously risk diverting seed dispersers from native plants and undermining long‐term ecosystem integrity.

## Graphical abstract

DNA metabarcoding of faecal samples from 21 frugivorous bird species in Gamboa, Panama, was used to assess how non‐native plants influence frugivory network structure. Non‐native species, though comprising only 28% of plants, reduced nestedness, increased connectance and modularity, and caused disproportionately high secondary bird extinctions while showing lower persistence during bird removals. These findings indicate that highly connected non‐native plants can function as key network connectors yet undermine long‐term ecosystem stability, with important implications for conservation and invasive species management.

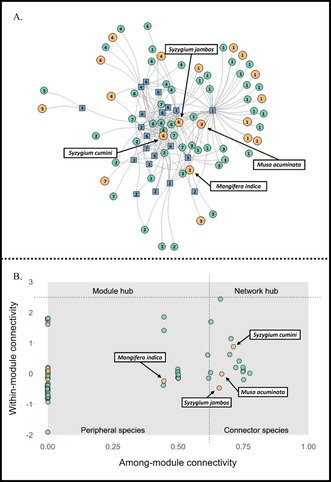

## 1 Introduction

Frugivory—the consumption of fruits by animals—is a crucial trophic interaction that links plants and animals in a mutually beneficial relationship (Jordano 1987). It plays a pivotal role in seed dispersal of both native and non‐native plants, which determines plant species distributions and population regeneration and can facilitate forest succession and promote ecosystem resilience (Escribano‐Avila et al. 2018). In particular, frugivorous birds can disperse seeds over long distances (Fell et al. 2023), helping to maintain plant genetic diversity across the landscape, and supporting ecosystem regeneration as well as crucial processes such as carbon sequestration (Camargo et al. 2022; Bello et al. 2024). However, today, more than 13,000 plant species, equivalent to ~4% of the extant global vascular flora, have become naturalised outside their native ranges as a result of human activity (Van Kleunen et al. 2015). These non‐native plants can alter the frugivory interaction networks that connect frugivores and plants, with knock‐on effects for ecosystem health and resilience (Costa et al. 2022; Dáttilo et al. 2023; Marciniak et al. 2024).

Frugivorous birds are key seed dispersers in tropical ecosystems and disperse more than 70% of tropical plant species (Sekercioglu 2006; Bradfer‐Lawrence et al. 2024). Frugivory interactions are shaped by trait matching between birds and plants, with bird beak morphology and body size determining which fruits can be physically consumed and dispersed (Rojas et al. 2022; Carlo et al. 2022). Similarly, plant functional traits—such as fruit size, seed mass, and plant height—play a complementary role by influencing fruit accessibility, attractiveness to frugivores, and the potential for successful dispersal, ultimately shaping the structure and *robustness* of frugivory networks.

Plant functional traits also shape species' roles within frugivory networks and may determine the success with which non‐native plants integrate into native communities. Seed size and mass constrain which frugivores can consume and effectively disperse propagules, with smaller seeds accessible to a broader range of frugivore body sizes (Muñoz et al. 2017). Plant height influences fruit visibility, accessibility across vertical forest strata, and the diversity of frugivore assemblages that visit fruiting plants, with taller plants often supporting more diverse and abundant frugivore communities (Schleuning et al. 2011; Durand‐Bessart et al. 2023). For non‐native plants establishing in tropical landscapes, these traits may act as filters determining integration success: species with trait combinations closely matching those of native plants may be more readily incorporated into existing frugivory networks, while those with divergent traits may either fail to establish or fundamentally alter network structure (Rojas et al. 2019; Vizentin‐Bugoni et al. 2021).

Non‐native plants can significantly alter foraging behaviour and interspecific competition among frugivores. When non‐native plants establish, temporary increases in food availability can shift foraging patterns among different animal groups (Hopson et al. 2020). This disruption may lead to preferential consumption of non‐native fruits, increasing seed dispersal for non‐native species and further promoting their spread (Grass et al. 2014). These changes in resource availability and foraging preferences can intensify competition among plant species for frugivore visitation, with non‐native plants potentially outcompeting native species for seed dispersal services. This may disadvantage native plant recruitment and alter the structure of plant communities within the ecosystem.

Beyond direct species interactions, non‐native plants can modify fundamental ecosystem processes by altering the physical environment and resource availability. Non‐native species often alter access to nesting sites, roosts, or soil nutrients, leading to shifts in species abundance and distribution (Weidenhamer and Callaway 2010; Nelson et al. 2017). Non‐natives may also introduce novel pathogens or parasites that affect local frugivores or plants. These can either directly reduce fitness or indirectly alter foraging behaviour (Young et al. 2017), ultimately compromising ecosystem resilience.

Given these complexities, we need an improved understanding of how non‐native plant species can impact frugivory networks in tropical ecosystems. To do this requires a comprehensive approach that considers direct and indirect interactions between frugivores and native and non‐native plants. Frugivory interactions can be described using species interaction networks, where the nodes correspond to the co‐occurring species and the links represent their interactions. This approach reveals which fruiting plant species provide food resources to which animal species and offers a unique window into the complex dynamics of ecosystems and the relationships that underpin their health and resilience (Jordano et al. 2011). Network metrics, such as *modularity*, *connectance* and *nestedness*, can quantify changes in network structure, providing insights into the resilience and stability of ecological interactions (Jordano 1987; James et al. 2012; Nordbotten et al. 2018). Network metrics can therefore be used to assess how changes in species composition—due to non‐native plant invasions—impact species interactions and broader ecosystem processes.

Understanding the dynamics of non‐native plants in frugivory networks is crucial for informing conservation and management strategies, particularly amid global biodiversity loss and invasive species challenges. Although several studies have examined non‐native plants in these networks, most focus on mechanisms of integration (Rojas et al. 2019; Vizentin‐Bugoni et al. 2021), context‐specific impacts such as seasonality or management interventions (Costa et al. 2022; Marciniak et al. 2024), or broad‐scale patterns across multiple networks (Dáttilo et al. 2023; Heleno et al. 2013). In contrast, our study explores the functional consequences of non‐native plants for the stability and resilience of a local frugivory network in a human‐modified landscape. Specifically, we examine (i) whether plant functional traits (seed size, seed mass, and plant height) differ between native and non‐native species and how non‐native plants are distributed across frugivorous bird diets; (ii) whether non‐native plants influence network structure and metrics of *nestedness*, *modularity*, and *connectance*; (iii) what structural roles non‐native plant species occupy within the network; and (iv) whether plant functional traits influence the *robustness* and resilience of the interaction network to sequential species removals.

## 2 Methods

**FIGURE 1:**
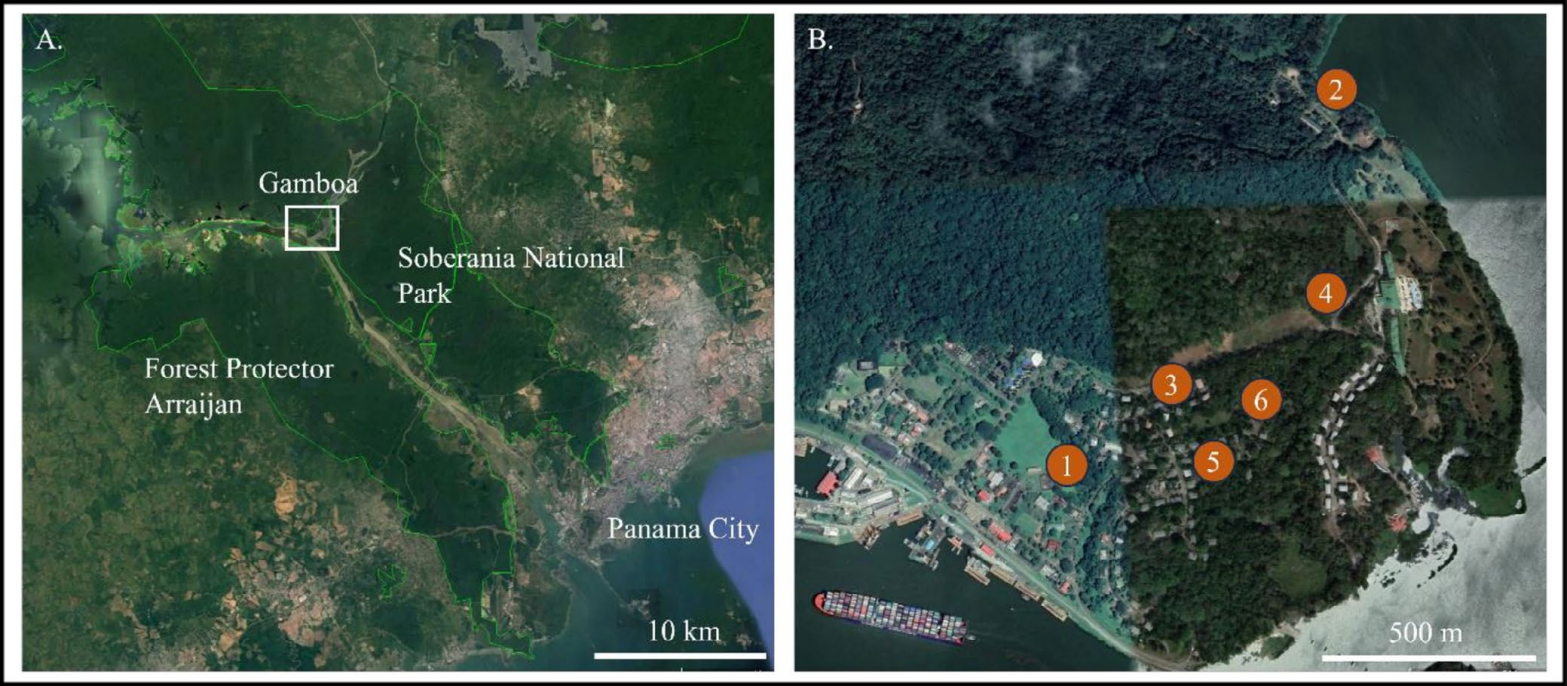
(A) Map of the location of Gamboa, in relation to Panama City and the two protected forests: Soberania National Park and Forest Protector Arraijan. (B) The town of Gamboa with numbers and circles representing the locations of mist netting for faecal sampling. Numbers correspond to those found in Table S1.

This study was conducted in and around Gamboa, a town along the Chagres River in central Panama, that is adjacent to Soberania National Park and across the Panama Canal from Forest Protector Arraijan (Figure 1A,B; −79.69707, 9.11624). The local forest is a mosaic of secondary forest (20–80 years old) and old‐growth lowland moist forest. The town contains residential areas with abundant planted street trees, gardens (often with non‐native plant species), forest patches and open grasslands. Gamboa's urban and peri‐urban areas feature significant human‐mediated plant assemblages, with many non‐native plant species having been introduced over the past 100+ years. These include intentionally planted ornamentals and agricultural plants. This introduced flora creates a landscape mosaic distinctly different from intact forest systems like Soberania National Park and nearby Barro Colorado Island (BCI), which is located only 16 km away and is predominantly old‐growth forest protected from significant human disturbance.

Central Panama has a tropical climate with a mean annual temperature of 28°C and a unimodal precipitation regime averaging 2226 mm annually, with a dry season from December to April and a wet season from May to November (Physical Monitoring Program, STRI n.d). The end of the dry season marks a critical period for fruiting tropical trees, with many species reaching peak fruit production in March and April before the onset of the wet season. To collect faecal samples for dietary analysis, birds were captured using mist nets between 13 January and 16 March 2022 at six locations near Gamboa (Figure 1B). These locations were chosen to reflect a range of environmental conditions and represent a gradient of human influence, from our most disturbed site within a garden of the residential area (site 3) to our least disturbed site (site 2) managed park land adjacent to continuous forest. of Central Panama.

### 2.1 Data Collection

To capture a representative sample of frugivorous birds inhabiting different ecological niches in the study site, we used nylon mist nets (6 × 12 m, mesh size 60 × 60 mm) placed at six, distinct locations around Gamboa. At each site, a single net was deployed at a height that matched the local vegetation structure, ranging from ground level to 15 m above the forest floor. This approach allowed us to sample frugivores foraging across different vertical strata—including the forest floor, understory, midstory, and canopy—each net targeted one height stratum per location, depending on the surrounding vegetation.

Mist nets were opened at the onset of daylight, 05:00 Eastern Standard Time (EST), to coincide with the activity period of many avian frugivores. Nets were operational until 10:30 EST, after which they were closed. Nets were reopened at 16:00 EST and closed again at 18:30 EST, just before dusk. Throughout the operational hours, each net was continuously monitored to ensure that any captured birds did not experience prolonged exposure to high temperatures or stress.

Captured birds were placed in small pet carry crates to collect faecal samples. The crates were modified to create a darkened environment to minimise stress for the birds. The crate floor was lined with clean paper for each individual to ensure no cross‐contamination between samples. Captured birds were held for a maximum of 20 min. Almost all individuals freely defecated within this timeframe. Faecal samples were collected from the crate floor and carefully transferred into a small, sterile plastic vial. To maintain the integrity of the collected samples, all vials were stored in a cooler containing ice packs and transported from the field site to the laboratory within 3 h of collection, then transferred to a −20°C freezer for long‐term storage.

All handling protocols were approved by both the Animal Welfare and Ethical Review Body (AWERB) at the University of Stirling and the IUCAC of the Smithsonian Tropical Research Institute. Our data collection methods were designed to prioritise the welfare of captured birds while obtaining faecal samples for our study. See Table S1 for the complete sample data.

### 2.2 DNA Extraction, Sequencing and Bioinformatics

DNA samples were extracted using the QIAamp PowerFecal DNA Kit (Qiagen), starting with 250 mg of material or the entire faecal pellet if it was less than that < 250 mg. An Illumina metabarcoding library was prepared to assess bird diets using a two‐step PCR approach. The first PCR (PCR1) was conducted in triplicate to amplify the ITS2 region with primers ITS2‐S2F and ITS2‐S3R (Chen et al. 2010) in a 10 μL reaction mix, consisting of 5 μL PCR buffer, 0.075 μL KAPA3G Plant DNA polymerase (Roche), 0.2 μL each of 10 mM forward and reverse primers, and 1 μL of DNA extract. Subsequently, a second PCR was performed to add the indexes in a 10 μL reaction, using 5 μL Platinum II Hot‐Start PCR Master Mix (Thermofisher), 1 μL each of 2.5 mM forward and reverse primers (with a unique index combination per sample), and 1 μL combined product of the PCR1 triplicates. PCR1 conditions included an initial denaturation at 95°C for 3 min, followed by 35 cycles of 95°C for 45 s, 56°C for 45 s, and 72°C for 45 s, with a final extension at 72°C for 5 min. Index PCR conditions involved an initial denaturation at 94°C for 3 min, followed by 6 cycles of 94°C for 45 s, 50°C for 60 s, and 72°C for 90 s, with a final extension at 72°C for 10 min.

PCR products were then purified using the Just‐a‐Plate 96 PCR Normalisation and Purification Kit (Charm Biotech) and combined into a single library. The final library was concentrated using Kapapure beads (Roche) at a 1X concentration. The library was sequenced on an Illumina Miseq using a 600‐cycle V3 sequencing kit.

Quality control of sequence data was performed using the DADA2 V1.16 pipeline (Callahan et al. 2016). Following a similar protocol to Clever et al. (2022), sequences were removed if they had more than two expected errors (maxEE = 2), at least one ambiguous nucleotide (maxN = 0), or at least one base with a high probability of erroneous assignment (truncQ = 2). Following visual inspection of base quality score plots, the forward sequences were trimmed to 260 base pairs, and reverse sequences were trimmed to 200 base pairs to remove low‐quality bases while maintaining adequate overlap for read pairing. Any sequences identified as chimeras were removed, and the final paired sequences were dereplicated and used to produce Amplicon Sequence Variants (ASVs). A total of 2727 ASVs were identified.

### 2.3 Taxonomic Assignment

Before assigning the ASVs to taxonomic identities, we first created a suitable reference database using two plant databases unique to Panama and a third global database. The first Panama‐specific database incorporated 19,663 sequences from 9393 plant species (Saltonstall et al. 2023), and the second was limited to 80 plant species found on Barro Colorado Island (Saltonstall et al. 2023; 9°9′42.36″N, 79°50′15.67″W) located approximately 16 km from our study site. Our study site includes many gardens and cultivated areas where many non‐native species are present. Hence, to allow for identification of non‐native species, we included a comprehensive list of world vascular plants as a third database which added 96,990 ITS2 sequences from 59,718 species (Banchi et al. 2020). We then subset this large database to include only those species found in Central America using the ‘World Checklist of Vascular Plants’ (POWO 2023) and ‘Tree Atlas of Panama’ (Perez and Condit 2023) to describe a comprehensive list of plant species present in Central America, both native and non‐native. We integrated the two local Panama databases and the World ITS2 database (subset to species found in Central America) to generate a database of 13,041 sequences from 5433 plant species.

To ensure that all reference databases were usable and could be successfully combined, taxonomic discrepancies among species were manually standardised following the taxonomy provided by *Plants of the World Online* (Kew), where inconsistencies in species classification were identified and adjusted manually to ensure consistency across databases. Reference sequences were analysed to determine duplicates across the different databases (95% similarity). Duplicated species were removed, and one identifier with the longest sequence length was retained per species. Our final reference library consisted of 5165 plant species with one sequence each. See Figure S1 for the complete decision workflow for creating our customised ITS2 reference database.

We assigned taxonomy to species level in all ASVs using our custom database and the RDP classifier implemented in the DADA2 pipeline. For any remaining unidentified ASVs, we prioritised those present in at least 1% of all samples for manual identification using the Basic Local Alignment Search Tool (BLAST) to find the most appropriate matches for the sequences (182 ASVs). We used a minimum query coverage of 95% and a minimum percentage identity of 90% to assign probable species. Finally, we filtered out any singletons, ASVs that occurred just once across all samples (68 ASVs), and a local botanist (co‐author DD) reviewed the final list of species to assess the likelihood of species occurrence.

### 2.4 Data Processing and Selection

To describe the plant taxa detected in bird faecal samples, we first conducted descriptive analyses based on the presence or absence of each plant taxon within a sample. We did not use relative read abundance (RRA) because variation in digestion rates and DNA degradation among bird and plant species can bias read counts. Instead, we calculated the frequency of occurrence (FOO)—the proportion of samples in which each plant species was detected—to identify the most prevalent taxa in the birds' diets.

For all subsequent analyses of species interaction networks, we used a subset of the data containing only fleshy‐fruited plant species, as these represent potential mutualistic partners involved in seed dispersal. This approach allowed us to focus specifically on interactions relevant to the frugivory–seed dispersal process. Binary matrices, as opposed to abundance matrices, were used to determine interaction network metrics.

### 2.5 Differences in Plant Traits

To determine whether differences in plant traits contributed to the changes seen across the network‐level metrics, we used Kruskal–Wallis tests to compare seed width, seed mass, and plant height between native and non‐native species as these data were not normally distributed. These traits represented mean values derived from multiple adult plants of each species, with the number of individuals used to calculate each mean varying across species depending on available data. The trait data were collected from various sources, primarily the NeoFrugivory database (Fuzessy and Pizo 2025), the Botanical Information and Ecology Network (BIEN) database (Maitner et al. 2018), the TRY Plant Trait Database (Kattge et al. 2011), and the SER Seed Information Database (SID 2023). Additional sources included the STRI online herbarium, Croat (1978) Flora of Barro Colorado Island and Bradfer‐Lawrence et al. (2024) Avian Frugivore‐Plant Interaction Networks of Barro Colorado Island.

### 2.6 Network Structure Comparisons

To understand how non‐native plant species affect the structure of the plant‐frugivore network, we calculated network‐level metrics using the *bipartite* package (Dormann et al. 2008). We analysed *nestedness* (the degree to which specialist species interact with subsets of species that interact with generalists), *connectance* (the proportion of possible interactions that are actually realised in the network), *modularity* (the tendency of species to form distinct interaction clusters or modules), and r*obustness* (the network's resilience to species extinctions). We calculated these metrics on the full network (all fleshy‐fruiting plant species; *n* = 69) and on a partial network (*n* = 50) where non‐native species were removed. We used *z‐scores* to evaluate the significance of each network‐level metric based on null‐model simulations, applying the *r2d* method for *connectance* and the *vaznull* method for *nestedness*, *modularity*, and *robustness* (Dormann et al. 2008). This approach allowed us to determine whether our observed network structures differed significantly from what would be expected by chance while accounting for differences in interaction frequencies and network sizes (Ulrich et al. 2009). For *robustness* specifically, we employed two removal methods: (1) degree‐based removal, sequentially removing the most highly connected species first (a stringent test of network vulnerability to targeted species loss), and (2) random removal as a control, where species were removed in random order to test network resilience to non‐selective disturbance. Each metric was assessed using 999 randomisations by comparing the observed values to the null distributions.

### 2.7 Species Interactions and Structural Roles

To assess species interactions, we performed two types of analysis that describe the uniqueness or similarity of the functional and structural roles between the native and non‐native species. First, to explore the similarity between the functional roles of the species, we quantified the overlap in the functional niche between native and non‐native species following Dehling and Stouffer (2018). In this framework, the functional role of a species is defined by the traits of its interaction partners rather than its own morphology—reflecting the idea that a species' ecological function is expressed through the traits of the resources it uses or provides within an ecological process. This approach allows meaningful comparisons of functional roles across taxa that participate in the same process, regardless of differences in their intrinsic morphology. To construct the functional trait space of a species, we used the traits of their interacting partner in a weighted Principal Component Analysis (PCA) via the *dudi.pca* function in the *ade4* package (R Version 4.2.2). All trait variables were standardised prior to analysis. We performed two separate PCAs: one for the Plant‐Bird Trait Space (using eight bird morphological traits, including weight and beak dimensions) and one for the Bird‐Plant Trait Space (using three plant traits: seed width, plant height, and seed mass). The frequency of observation (FOO) for each interaction, divided by 100, was applied as the row weight to ensure frequent interactions defined the realised niche space. Niche overlap was then visualised (Figure S4) by plotting the species' scores on the first two axes and drawing confidence ellipses around the native and non‐native groups.

Second, to explore the structural role that individual fleshy‐fruiting plant species play in the network, we identified their location within the network by categorising them as peripheral species and connector species. Peripheral species occupy the edges of the interaction network, engaging mainly within their own module, whereas connector species link different modules together and facilitate network cohesion. We followed the framework of Olesen et al. (2007), which classifies species based on two metrics: the within‐module degree (z), describing how well a species is connected to other species within its own module, and the among‐module connectivity (c), indicating how evenly a species' links are distributed across modules. Species with low z (< 2.5) and low c (< 0.62) values were classified as peripheral, while species with low z (< 2.5) but high c (> 0.62) values were classified as connectors.

### 2.8 Network Robustness to Species Extinctions

Network *robustness* was assessed through two extinction simulation models: (1) *Bird Removal Scenario*—evaluating plant persistence following sequential bird removals, and (2) *Plant Removal Scenario—*measuring total bird secondary extinctions following sequential plant removals. Code from Vizentin‐Bugoni et al. (2022) was modified to implement rewiring, enabling species to form new interactions after partner removal. Rewiring was a two‐step process: species first attempted new interactions with a probability proportional to the number of existing connections (*degree*) of potential partners, and then successfully formed new links based on trait matching between bird beak volume and seed width. Beak volume was calculated using beak length, depth, and width because beak gape data was not available for all species (Tobias et al. 2022).

For the *Bird Removal Scenario*, 1000 simulations were performed, sequentially removing bird species in order of decreasing *degree* (number of interactions), highest first, until 16 of 21 species (75%) were removed. This threshold represents a severe but not complete network collapse, allowing identification of plant species most likely to persist despite substantial frugivore loss while maintaining sufficient network structure for analysis. The degree‐based extinction method was selected because it targets the most connected species first—those with the highest number of interactions—which are often the most ecologically influential. This approach constitutes a biologically meaningful and stringent test of network *robustness*, as removing highly connected species causes greater disruption to network structure and functioning. Plants were considered extinct when they lost all frugivore partners and had no successful rewiring. The plant species remaining after each simulation were recorded.

For the *Plant Removal Scenario*, we conducted 1000 simulations, each sequentially removing all plant species in order of decreasing *degree*. After each plant removal, bird species that lost all plant partners and failed to rewire were recorded as secondary extinctions, representing frugivores that would theoretically lose all food resources in the network despite attempts to find alternative partners. The cumulative number of secondary bird extinctions triggered by each plant species removal across all replications was recorded.

Negative binomial regression models with log link functions were implemented to identify characteristics associated with network persistence and secondary extinctions. For the *Bird Removal Scenario* model, the response variable was the frequency of plant survival into the final five extinction steps across bird removal simulations (0–1000). Plant characteristics (Native vs. non‐native, woody vs. herbaceous, seed width, seed mass, plant height) served as explanatory variables. For the *Plant Removal Scenario* model, the response variable was the cumulative total of secondary bird extinctions each plant species caused across simulations. Plant characteristics served as explanatory variables, with *degree* included as a predictor in all models due to its expected strong influence on extinction outcomes. Despite seed width showing significant differences between groups in Kruskal–Wallis tests, seed width was excluded from final models due to collinearity with seed mass. Models incorporating seed mass yielded substantially better AIC values than those using seed width. Interaction terms between plant nativeness and other characteristics were included to test whether trait effects differed between native and non‐native species.

Model selection was based on AIC scores and residual diagnostics. All continuous predictor variables were centred and scaled prior to analysis. All statistical analyses were conducted in R version 4.4.1 (R Core Team 2022). Full model outputs, including *z*‐tests, *p*‐values, and summary tables, are provided in Tables S2 and S3.

## 3 Results

Of the 2727 ASVs initially identified, 68 singletons were removed from subsequent analysis, resulting in a final dataset of 2659 ASVs. Taxonomic assignment identified 1725 ASVs (64.9%) at the species level, while 341 (12.8%) were assigned only at the genus level and 197 (7.4%) only at the family level. The remaining 396 ASVs (14.9%) could not be reliably assigned below the family level.

### 3.1 Composition of Faecal Samples

**FIGURE 2:**
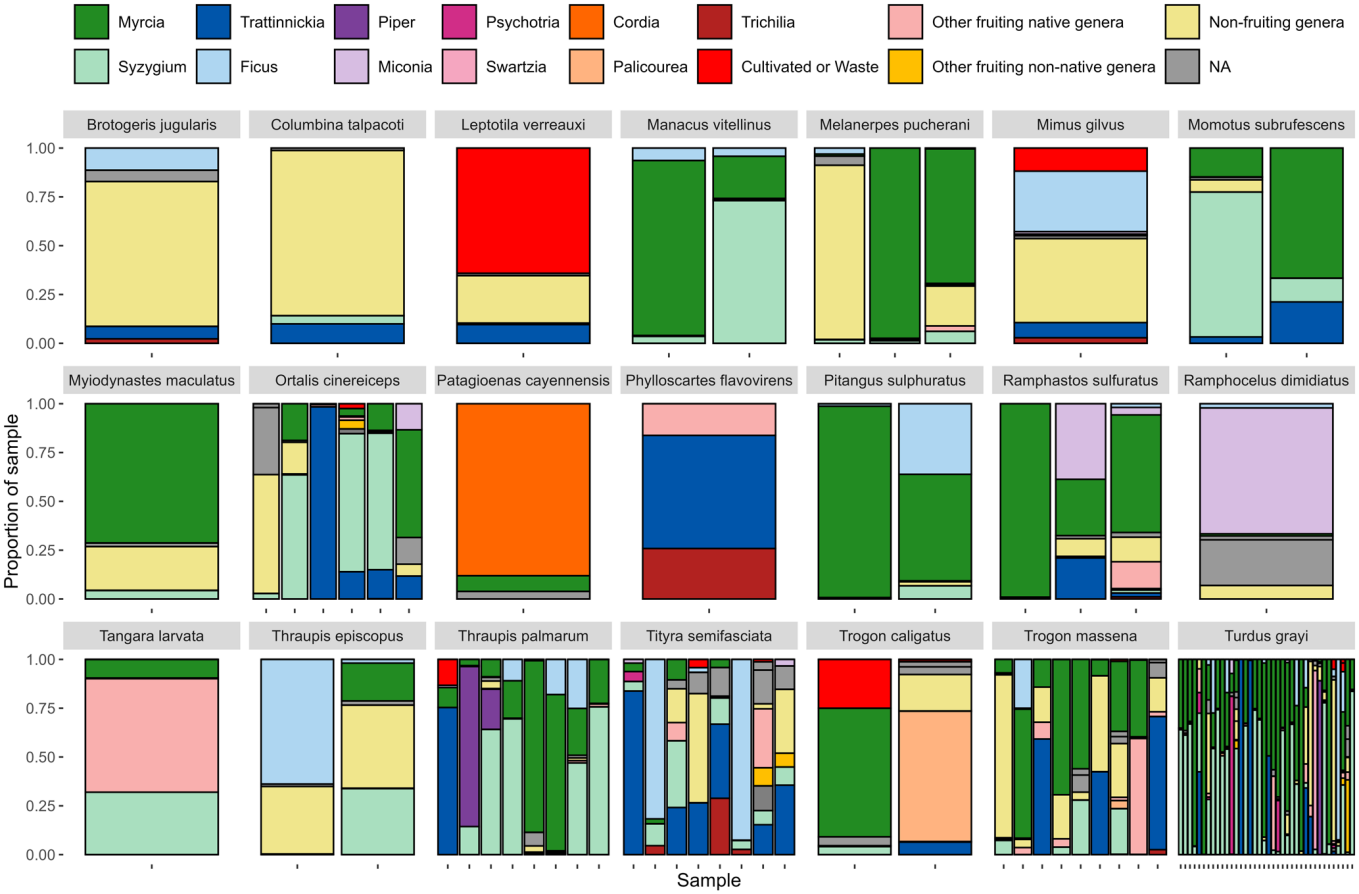
The proportion of identified plant genera in each individual faecal sample, grouped by bird species (*n* = 21 species). Each bar represents a single faecal sample. The 11 most abundant plant genera are shown individually by colour, while less common genera are grouped into ecological categories. ‘Other fruiting native genera’ includes all native genera with fleshy fruits not among the top 11. ‘Other fruiting non‐native genera’ represents non‐native genera with fleshy fruits. ‘Non‐fruiting genera’ includes plants lacking fleshy fruits, representing consumption of vegetative plant material rather than fruits. ‘Cultivated or Waste’ comprises species likely associated with garden waste (*Cicer arietinum, Daucus carota, Allium cepa*) or cultivated species (*Musa acuminata, Coffea arabica, Mangifera indica, Passiflora edulis*). ‘NA’ indicates the proportion of each sample that could not be identified to genus level.

We recorded a total of 127 plant species across all faecal samples: 95 native and 32 non‐native, 69 fleshy‐fruited and 58 non‐fleshy‐fruited, and 95 woody compared to 32 non‐woody species. On average, all bird species consumed a higher proportion of fleshy‐fruited, native and woody plants. The exceptions were the *Ramphocelus dimidiatus* , *Brotogeris jugularis* , *Columbina talpacoti* and *Leptotila verreauxi* which consumed more non‐fleshy‐fruited plants, with *Columbina talpacoti* also consuming more non‐native species (Table S2). Three non‐native species— *Cicer arietinum* (chickpea), *Daucus carota* (carrot), and *Allium cepa* (onion)—were likely consumed from garden food waste. Additionally, cultivated non‐native plants such as *Musa acuminata* (banana), *Coffea arabica* (coffee), *Mangifera indica* (mango), and *Passiflora edulis* (passionfruit) may have been acquired from either garden waste or fruiting plants in the local landscape. Nine bird species included these cultivated species in their diets: *Turdus grayi (Passiflora edulis*, *Mangifera indica* , *Musa acuminata* ), *Tityra semifasciata* ( *Allium cepa* , *Coffea arabica* ), *Trogon caligatus* ( *Mangifera indica* , *Daucus carota* ), *Trogon massena* ( *Coffea arabica* ), *Ortalis cinereiceps* ( *Cicer arietinum* ), *Leptotila verreauxi* ( *Daucus carota* ), *Pitangus sulphuratus* ( *Musa acuminata* ), *Thraupis palmarum* ( *Musa acuminata* ), and *Mimus gilvus* ( *Mangifera indica* ) (Figure 2).

The number of unique plant species detected per bird species ranged from three for *Phylloscartes flavovirens* (*n* = 1 sample) to 71 for *Turdus grayi*, (*n* = 38 samples), with a mean of 15 plant species (Table S2). The number of unique plant species per faecal sample ranged from one for *Turdus grayi* to 22 for *Trogon massena* , with a mean of eight. The most common plant species detected were *Myrcia sylvatica, Syzygium cumini and Myrcia guianensis* (all from the *Myrtaceae* family), found in 79%, 69% and 64% of all samples, respectively. The remaining plant species appeared in fewer than 50% of samples, with 33 species detected only once. *Fabaceae*, *Rubiaceae*, *Poaceae*, *Arecaceae*, and *Euphorbiaceae* were the most common families, each with 12, 12, 9, 8, and 8 species represented, respectively. However, *Myrtaceae* emerged as the most abundant family found in all samples.

### 3.2 Trait Comparisons and Network‐Level Metrics

No significant difference was found in average plant height and seed mass between native and non‐native plant species. However, non‐native plant species had significantly larger seed widths compared to native species (Kruskal–Wallis—*p* < 0.05; Figure S2).

**FIGURE 3:**
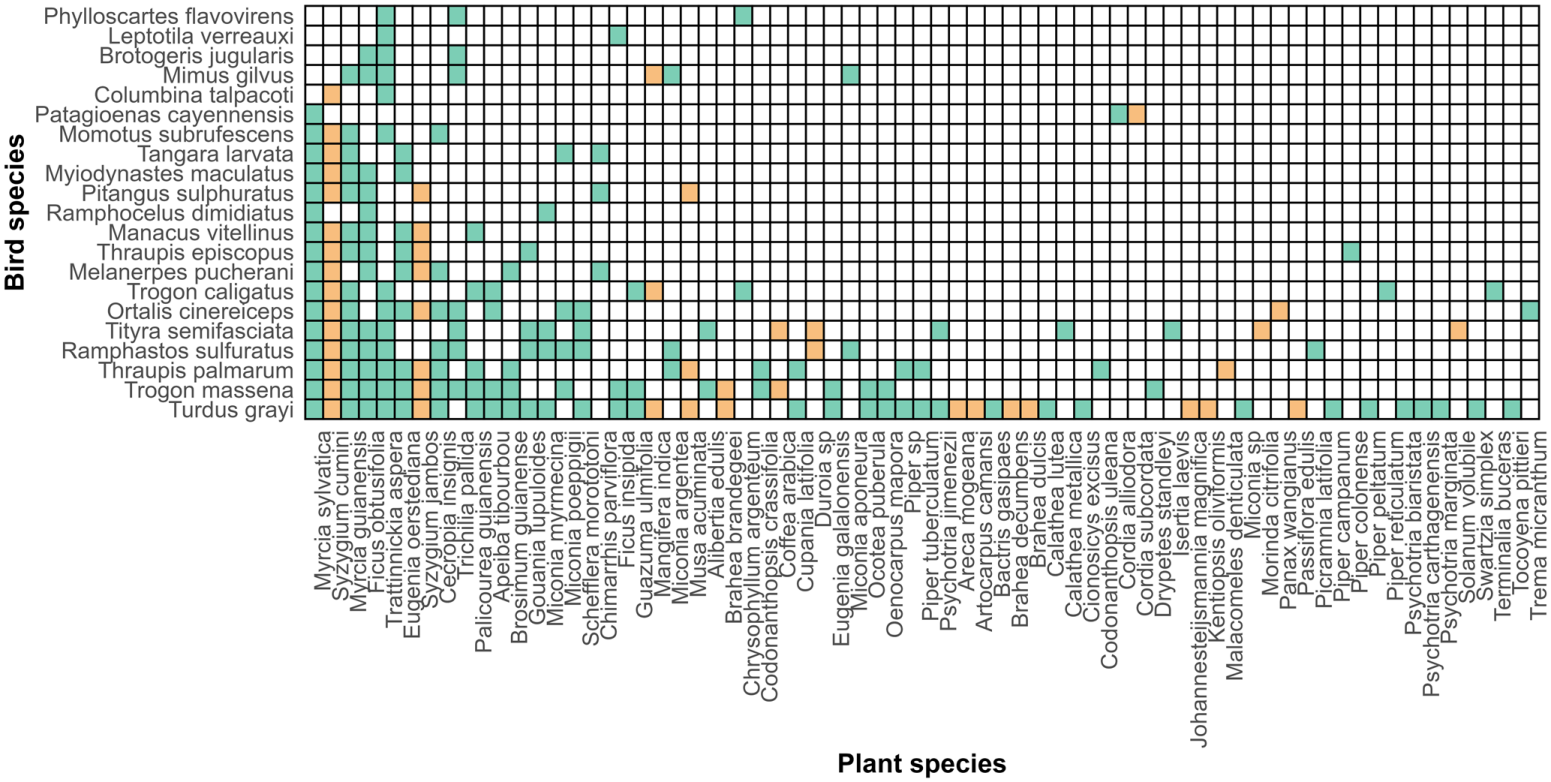
Matrix of interactions in the Gamboa avian frugivore‐fruiting plant network. Bird species (*n* = 21) as rows, ordered top to bottom with increasing numbers of samples, and fleshy‐fruited plant species (*n* = 69) as columns, ordered left to right with decreasing number of connections. Green squares represent native plant species, and orange squares represent non‐native species.

The fleshy‐fruiting plant network displayed a moderately nested structure, with a *nestedness* value of 41.7 (*z* = 5.53; *p* < 0.001; Figure 3) in the full network and 44.5 (*z* = 5.84, *p* < 0.001) in the native‐only network (Table 1). These high *z*‐scores indicate that the observed *nestedness* is significantly greater than expected by chance. To help interpret these values, note that a network with no *nestedness* would approach zero, whereas values approaching 100 would represent a perfectly nested network.

**TABLE 1:** Changes in network metric values and individual *z*‐scores between the full frugivory network (fleshy‐fruiting species only) and the network excluding non‐native plant species. Metrics include *connectance* (C), *nestedness* (NODF), *modularity* (Q), and *robustness* (R), both higher level and lower. *Robustness* includes removal methods of *degree* and random. The *z*‐scores in bold represent the metrics that differed significantly from what would be expected by chance.

	Full network		Native network	
	Metric value	*z*‐score	Metric value	*z*‐score
Connectance	0.145	**10.59**	0.155	**9.87**
Nestedness	41.7	**5.53**	44.5	**5.84**
Modularity	0.378	**2.434**	0.363	0.871
*Robustness method = degree*				
Robustness.HL	0.753	**−2.697**	0.715	**−2.316**
Robustness.LL	0.520	**−4.27**	0.538	**−3.494**
*Robustness method = random*				
Robustness.HL	0.931	−0.213	0.905	−0.305
Robustness.LL	0.760	−1.186	0.771	−0.931

*Connectance* was elevated in both the full and native‐only networks compared to null expectations (*z* = 10.59, *p* < 0.001 for the full network; z = 9.87, *p* < 0.001 for the native network; Table 1), indicating a higher‐than‐expected proportion of realised interactions. *Modularity* was moderate in both networks (0.378 in the full network and 0.363 in the native‐only network; Table 1), indicating some degree of compartmentalisation, where groups of species interact more frequently within modules than between them. However, when compared to null models, only the *modularity* of the full network was significantly greater than expected by chance (*z* = 2.434, *p* < 0.05), while the native network did not differ significantly from the null expectation (*z* = 0.871, *p* > 0.05).

The structure of the network was shaped by a skewed distribution of interactions, where a small number of plant species accounted for a large share of bird visits, while many others were infrequently visited (Figure 3). This pattern contributed to the observed *nestedness*. Fleshy‐fruiting plants accounted for the majority of interactions, and non‐native species were disproportionately involved. The average number of interactions for native species was 3.26, while for non‐native species it was 2.47. A Wilcoxon rank sum test indicated that the difference between native and non‐native species' interactions was not significant (*p* = 0.1257). However, the two *Syzygium* species ( *Syzygium cumini* and *Syzygium jambos* ), which had much higher interaction counts (15 and 8 respectively), exhibited a significant difference in the number of interactions compared to the other non‐native species (*p* = 0.01). These two non‐native species exhibited some of the highest degrees of connectivity in the network.

The removal of non‐native plant species drove changes in network *robustness* under targeted (degree‐based) extinction (Table 1). Both *Robustness.HL* (birds) and *Robustness.LL* (plants) were significantly lower than expected by chance in both the full and native‐only networks, with stronger deviations in the full network (*z* = −2.697, *p* < 0.05 for HL; z = −4.270, *p* < 0.001 for LL) compared to the native network (*z* = −2.316, *p* < 0.05 for HL; *z* = −3.494, p < 0.001 for LL). The removal of non‐native species weakened both plant‐level and bird‐level *robustness* under *degree* extinction. Under random extinction, however, *robustness* values were high across all networks and not significantly different from null expectations.

### 3.3 Modularity and Species Roles

**FIGURE 4:**
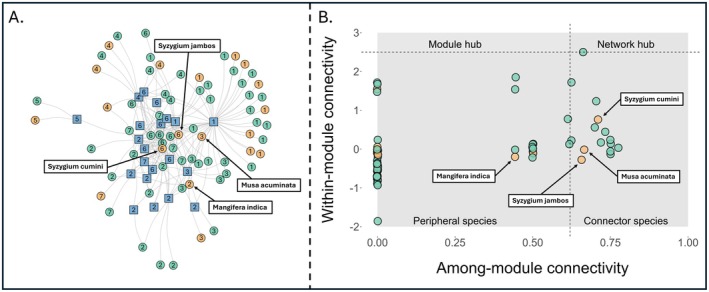
(A) Bipartite frugivory interaction network of fleshy‐fruited plants and frugivorous birds. Blue squares represent frugivorous bird species. Dots represent fruiting plant species, with green indicating native plants and orange indicating non‐native plants. Links between nodes represent observed frugivory interactions. Numbers in nodes represent the distinct modules each species are part of. (B) Module roles of fruiting plants within the Gamboa frugivory network. The network consists of 54 peripheral species and 15 connector species. Non‐native species are denoted by orange dots.

The fleshy‐fruiting plant network naturally partitioned into seven distinct clusters of nodes (modules)—each representing either a bird or plant species—where the internal interactions within these clusters are stronger than those between species across clusters (distinct module clusters are represented by the values inside the nodes; Figure 4A). Non‐native plant species showed a highly clustered distribution, with 12 of the 19 total non‐native species (63%) concentrated in just two modules containing 8 and 4 non‐native species in Modules 1 and 4, respectively (Figure 4A). Peripheral species, exhibiting low within‐ and among‐module connectivity, comprised 54 plant species in the network. A total of 15 plant species were identified as connectors (with among‐module connectivity *c* > 0.62), including three non‐native species: *Syzygium jambos* , *Syzygium cumini* , and *Musa acuminata* (Figure 4B). No species were classified as module hubs or network hubs, although *Trattinnickia aspera* is near being classified as a network hub (Figure 4B).

### 3.4 Influence of Plant Characteristics on Network Robustness

Simulations of the *Bird Removal Scenario* revealed that six plant species consistently persisted through to the final five removal steps across all 1000 replications. Among these were two non‐native species, *Syzygium cumini* and *Syzygium jambos* , which remained in the network for every replication even after 75% of bird species (16 out of 21) had been removed.

**FIGURE 5:**
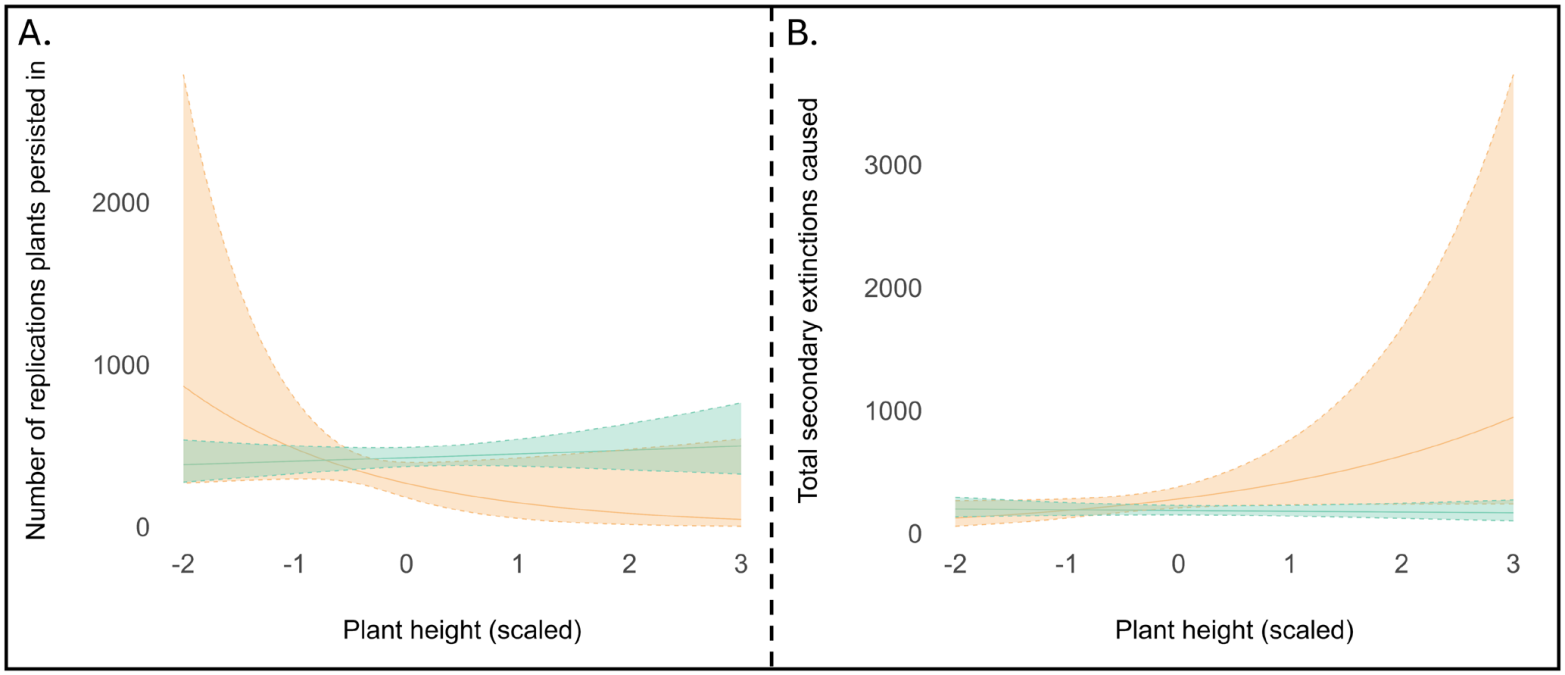
(A) Interaction between plant nativeness and height on species persistence during simulated extinction replications. Persistence reflects the number of replications that a plant species survived up until the last 5 bird removals. The interaction was non‐significant (*p* = 0.08; see Table S3). (B) Interaction between plant nativeness and height on cumulative secondary extinctions during simulated species removals. Total secondary extinctions represent the total number of bird species lost following the removal of each plant species over all 1000 replications. The interaction was statistically significant (*p* < 0.05; see Table S4). For both figures, native plants are represented by green and non‐native plants by orange. Lines represent model predictions from the negative binomial regressions, with shaded areas indicating 95% confidence intervals.

Results from the negative binomial model showed that native plant species had significantly higher persistence than non‐native species (estimate ± SE = 0.466 ± 0.215, *p* < 0.05; Table S3). *Degree*—the number of interactions per species—also had a significant positive effect on persistence, with more connected plants surviving longer into the extinction sequence (estimate ± SE = 0.393 ± 0.069, *p* < 0.05). An interaction between nativeness and plant height was not statistically significant (estimate ± SE = 0.640 ± 0.376, *p* = 0.08), but the data suggested a tendency for persistence to increase with height among native species while decreasing with height in non‐native species (Figure 5A). Neither plant height nor seed mass alone significantly predicted persistence (*p* > 0.05; Table S3).

### 3.5 Drivers of Secondary Extinctions

Using simulations from the *Plant Removal Scenario*, we found that, on average, the removal of a single non‐native species resulted in a higher number of secondary bird species extinctions per simulation (non‐native: 0.371 ± 0.133 SD; native: 0.279 ± 0.051 SD; Wilcoxon rank sum test, *W* = 261,367, *p* < 2.2e‐16). Non‐native removals also exhibited substantially greater variability in their impacts compared to native species, as evidenced by the larger SD (Figure S3).

The negative binomial model examining total secondary extinctions showed that the removal of native plant species caused significantly fewer secondary extinctions compared to non‐native species removal (estimate ± SE = −0.414 ± 0.146, *p* < 0.05; Table S4). Plant height had a marginally significant positive effect, with taller plants tending to cause more secondary extinctions (estimate ± SE = 0.405 ± 0.209, *p* = 0.05; Table S4). A significant interaction was found between nativeness and plant height, where secondary extinctions slightly decreased with increasing height in native species but increased with height in non‐native species (estimate ± SE = −0.438 ± 0.223, *p* < 0.05; Figure 5B; Table S4). *Degree* had a strong negative effect on the total number of secondary extinctions, with more connected species causing fewer extinctions (estimate ± SE = −1.046 ± 0.161, *p* < 0.05; Table S4). Seed mass was not a significant predictor of secondary extinctions, either on its own or in interaction with nativeness (*p* > 0.05; Table S4).

## 4 Discussion

Non‐native plants, particularly *Syzygium* species, play a structurally important role in the local tropical frugivory network of Gamboa, Panama during the dry season. While native plants found in the local landscape dominated the diet of most frugivorous birds, the presence of 32 non‐native plant species across bird diets reflects the successful integration of non‐native plants into the ecological community. Some of these plants have been established in Gamboa for decades, suggesting that what was once dietary plasticity may now represent established feeding behaviours. We found that non‐native species provide additional food resources for frugivores yet simultaneously disrupt the natural structural properties that characterise native plant‐bird interactions. In general, the inclusion of non‐native plants decreased the network's *robustness* to secondary extinctions, whilst the removal of these species altered other key network‐level metrics, suggesting they modify network structure in potentially destabilising ways. Non‐native plants caused disproportionately more secondary bird extinctions despite comprising only 28% of fruiting plant species in our system. This paradoxical role, where some non‐natives serve as important food resources and network connectors while others undermine network stability, highlights the complex conservation challenges presented by novel plant‐frugivore interactions in human‐modified landscapes (Traveset and Richardson 2014; Vizentin‐Bugoni et al. 2019).

### 4.1 Non‐Native Plants Alter Network Structure Through Reduced *Nestedness* and Increased *Connectivity*

The structural role of non‐native plants becomes evident when comparing network‐level metrics between full and native‐only networks. The presence of non‐native species is associated with reduced *nestedness* but increased *connectance*, indicating that these species modify key structural features of the interaction network. Although the full network showed slightly lower *nestedness* than the native‐only network (41.7 vs. 44.5), both networks demonstrated significant *nestedness* structure (*z* > 5.5, *p* < 0.001), suggesting relatively strong hierarchical organisation in both cases. However, the modest reduction in *nestedness* when non‐native species are included may indicate a subtle decrease in interaction redundancy, potentially reducing network resilience (Bastolla et al. 2009; Mello et al. 2011). One example from our network is *Cordia subcordata* , a non‐native plant that, in this dataset, was consumed exclusively by the Pale‐vented Pigeon ( *Patagioenas cayennensis* )—a frugivore with few interactions that shares just one mutual partner with the network's most connected frugivores. This example of a unique link disrupts the typical nested pattern, where specialists usually interact with subsets of generalist partners. Conversely, the higher *connectance* in the full network reflects the addition of non‐native species and their interactions. However, many of these new connections are low‐quality and isolated—nine non‐native plant species were consumed by only one bird species—contributing to the overall number of realised links while also increasing the number of possible ones without strengthening overall network integration. These findings support the idea that, while non‐native plants may increase species richness and the number of connections, they can simultaneously weaken certain aspects of ecological network structure by adding interactions that may be isolated or poorly connected to the broader community (Darosci et al. 2017).

In contrast, the increase in *modularity* further indicates that non‐native species play a role in shaping and maintaining distinct functional groups within the network (Vizentin‐Bugoni et al. 2021). Following Dehling and Stouffer (2018), niche overlap analysis (Figure S4) reveals an asymmetrical interaction pattern: non‐native plants occupy a restricted subset of plant trait space, suggesting that only plants with specific trait combinations successfully integrate into the frugivore network. However, the bird community shows considerable flexibility, with similar bird trait profiles interacting with both native and non‐native plants. This pattern indicates plant selectivity rather than bird selectivity as the primary driver of network structure, where the establishment success of non‐native plants is constrained by their functional trait compatibility with the existing bird community. While this clustering pattern shares structural similarities with what D'Antonio and Dudley (1993) termed ‘invader complexes,’ our network represents a different ecological scenario, as it contains non‐native plants grouped within modules but lacks non‐native frugivores in our sampling. One module in particular, Module 1 (Figure 4A), contains a large cluster of non‐native plant species. These species are grouped together because they share similar interaction patterns, suggesting they may possess common traits that make them attractive or accessible to a specific subset of frugivorous birds. This could alter the interaction dynamics of that module, making it more distinct compared to others. Three of the network connector species are non‐native (two *Syzygium* species; Figure 4B), and their high connectivity between modules is crucial for linking different modules with one another, influencing the flow of resources and interactions across the network.

The structural role of *Syzygium* species as connector nodes in the network may have important ecological consequences. Their ability to bridge modules and maintain interaction flows across otherwise distinct groups suggests a functional advantage that could reinforce their ecological success. *Syzygium cumini* is a Category 1 invasive plant in Florida, USA and a Category 3 invasive plant in South Africa, and is also becoming invasive across several Pacific islands, especially in the Cook Islands (Global Invasive Species Database 2025). According to local forest inventories by CTFS‐ForestGeo in Panama (Condit et al. 2016), *Syzygium* species have been documented in 19 of 186 surveyed plots across Panama, with one establishment just 6 km from our study site. This local presence, combined with global invasion patterns, suggests active spread is already occurring. Similarly, Avalos et al. (2006) documented the overwhelming dominance of *S. jambos* in understory communities of premontane forests in Costa Rica, where it comprised 51% of 1285 sampled plants. If these *Syzygium* species are fruiting during periods when native plants are not, they could monopolise the attention of frugivores, potentially accelerating their spread and dominance in new environments within the region. This clear dietary shift may have adverse consequences for local native plant‐frugivore mutualisms. If native and non‐native plants fruit synchronously, competition for seed dispersers could divert frugivores away from native species, ultimately reducing native plant recruitment across the landscape (Traveset et al. 2012; Heleno et al. 2013). Conversely, if non‐native plants fruit outside the primary native fruiting season, they may attract a high number of seed dispersers, facilitating their further spread into the local environment (Vergara‐Tabares et al. 2018).

### 4.2 Bird and Plant Characteristics That Influence Secondary Extinctions

Extinction simulations for both bird and plant species revealed a complex picture regarding the role of non‐native plants in network stability. When plants were removed, non‐native species contributed disproportionately high rates of secondary extinctions among birds. However, when birds were removed, non‐native plants showed lower persistence compared to native species, indicating greater vulnerability to the loss of their avian partners. This finding creates a paradox: while some non‐native plants have a disproportionately high impact on network stability—causing more secondary bird extinctions when removed—others are themselves at greater risk during bird loss, showing reduced persistence compared to native species. Some non‐native species that act as connector nodes—such as *Syzygium* species—clearly support both generalist and specialist frugivores, helping integrate the network by linking distinct functional groups of birds together. These results indicate that non‐native plants, as a group, have significantly larger impacts on network stability than native plants. This effect was driven primarily by a small number of highly connected non‐native species (particularly *Syzygium* species), while the greater variability in non‐native impacts suggests their effects on network stability are less predictable than those of native species. However, this integrative role is not consistent across all non‐native plants, as nine non‐native species were connected to a single bird species. This mix of highly connected and poorly connected non‐native species likely contributes to the observed reduction in *nestedness* in the full network and explains why non‐native species may have become important food resources for some frugivores without fully integrating into the broader network. This mismatch between ecological importance and structural integration may constitute what can be described as an evolutionary trap (Schlaepfer et al. 2002; Robertson et al. 2013). Some birds may have come to rely on non‐native plants that appear abundant or seasonally dependable, yet these plants are inherently more vulnerable to species removal. If these non‐native plants are lost, the birds that have become dependent on them could suffer disproportionately, despite the short‐term stability they seem to offer.

We identified plant height as a particularly influential characteristic—taller plants, especially non‐natives, triggered more secondary extinctions when removed from the network. This mirrors findings from tropical forest systems showing that taller species play important roles in frugivore networks. For example, Schleuning et al. (2011) found that fruit crop size and frugivore visitation rates increased with plant height, suggesting that taller plants may offer greater resources and attract more mutualists. Similarly, Raoelinjanakolona et al. (2023) observed that key connector and hub species in Madagascan rainforests were tall canopy trees. Taller, late‐successional species often support a broader range of frugivores due to their accessibility and consistent fruit availability across vertical strata (Durand‐Bessart et al. 2023). The effect of height among non‐native plants may reflect a selection bias in the invasion process, where species of greater stature are more likely to successfully establish. This is supported by findings that invasive species tend to be taller than non‐invasive exotics, suggesting that plant height is a key trait linked to invasion success (Waddell et al. 2020; Gallagher et al. 2015). In this sense, the non‐native plants in our network likely represent a non‐random subset of species with the capacity to dominate vertical space and attract diverse frugivore assemblages.

This stature characteristic showed a marginal, contrasting effect in our persistence model: taller non‐native plants were less likely to persist in the network during sequential bird removals. While tall non‐native plants can support many frugivores, these plant species often rely on a limited subset of bird species for effective seed dispersal, creating an imbalanced mutualism. Taller plants likely depend on specific larger‐bodied, canopy‐dwelling frugivores capable of handling larger fruits and dispersing larger seeds (Thiel et al. 2021). Such specialised dispersers may be more sensitive to disturbance or have limited capacity to rewire interactions when faced with ecosystem changes (Uezu et al. 2005; Newbold et al. 2013), leading to this marginal significance we see in the model.

### 4.3 Implications of Non‐Native Plant Species for Local Frugivores and Native Plants

The integration of non‐native species from anthropogenic food sources, such as *Cicer arietinum* (chickpea), *Dacus carota* (carrot), and *Allium cepa* (onion), into frugivore diets highlights the adaptability and resilience of frugivores to environmental changes. Species typically associated with forest habitats, such as the *Tityra semifasciata* and *Trogon caligatus* also consumed plant species linked to household waste. This suggests that some forest‐dwelling birds may venture outside their preferred habitats to access easily available and reliable food sources. Similar patterns have been reported in urban and peri‐urban environments, where gardens within a forest reserve in Sri Lanka (Perera et al. 2017) supported the highest avifaunal diversity and greater bird richness. In fact, six of the nine species found consuming household waste were also in the top seven species with the most diverse diets, suggesting flexibility is associated with the consumption of novel food sources. This behaviour aligns with observations from other ecosystems, where birds demonstrate ecological flexibility by incorporating anthropogenic food sources into their diets (Ottoni et al. 2009). For example, parrot species such as the Scarlet Macaw ( *Ara macao* ), Golden‐capped Parakeet ( *Aratinga auricapillus* ), and Blue‐winged Parrotlet ( *Forpus xanthopterygius* ) have been observed feeding extensively on cultivated non‐native plants across human‐altered landscapes in Costa Rica and Brazil (Matuzak et al. 2008; Silva and Melo 2013).

Our findings from this human‐modified landscape provide an interesting contrast to studies from more intact forest systems like Barro Colorado Island (BCI). Nine bird species in our study are also present in the BCI dataset, where they are known to feed on native plant genera including *Miconia*, *Piper*, *Cecropia*, *Ficus*, and *Palicourea* (Bradfer‐Lawrence et al. 2024). However, we found that eight of these nine species have more diverse diets in Gamboa compared to BCI, consuming at least twice as many plant species—and in the case of *Trogon massena* , nearly five times as many (BCI: *n* = 5; Gamboa: *n* = 23), with four of these species being non‐natives. While methodological differences may contribute to this pattern—our study utilised DNA metabarcoding of faecal samples while BCI data come primarily from observational feeding surveys—the broader dietary breadth in Gamboa likely reflects the integration of non‐native plants and anthropogenic food sources. Despite these differences in diet breadth, we also found striking similarities in certain structural aspects of the networks. For instance, small‐seeded plants comprise 65% of plant species in our Gamboa network and 66% in the BCI system. Similarly, *Miconia* species serve as important food resources in both networks, feeding approximately half of the frugivorous bird species in each system. These consistencies suggest that certain fundamental aspects of tropical frugivory networks persist despite habitat modification and the integration of non‐native species.

### 4.4 Caveats and Limitations

While our study provides valuable insights into the dietary composition, network structure, and role of non‐native plant species within the frugivore community of this part of Central Panama, we acknowledge some limitations that may influence the interpretation of our findings. The uneven and relatively small sample sizes across bird species necessitate cautious interpretation of our results. Some species were represented by numerous faecal samples (e.g., *Turdus grayi* with 38 samples), while others had minimal representation (nine bird species with just one sample each), potentially introducing sampling bias and limiting our ability to draw comprehensive conclusions about interspecific dietary patterns.

Further, our methodology provides only a temporal snapshot of these interactions, capturing a specific moment in a dynamic ecological system; we sampled for 2 months during the dry season. Frugivore diets can exhibit significant seasonal variability, influenced by fruit availability, phenology, and resource abundance (Loiselle and Blake 1991; Pizo et al. 2021). The broad dietary breadth we observed, particularly among some of the more generalist species, may reflect adaptive foraging strategies during a period when certain native fruits are scarce. The consumption of non‐native plants, particularly *Syzygium* species which fruits in the dry season, could represent an important seasonal resource buffering the effects of natural phenological cycles. Without wet season sampling, we cannot determine whether the integration of non‐native species represents a year‐round dietary shift or a seasonal adaptation to resource scarcity. Future studies would benefit from expanding the temporal scope to include peaks and troughs in fruit production and increasing the spatial coverage to improve our understanding of the complex web of frugivorous interactions within this landscape.

A final caveat of our study is that the metabarcoding approach detects consumption but cannot fully distinguish between mutualistic seed dispersal and seed predation. Although the primary role of some bird groups (e.g., doves and parrots) is seed predation, a proportion of the seeds they consume or handle can survive gut passage or be regurgitated intact, contributing to seed dispersal (Blanco et al. 2015; Tella et al. 2016; Mokotjomela et al. 2016). Given that our analyses are based on presence/absence data, their inclusion reflects valid frugivory interactions and the potential for seed dispersal within the network. Therefore, while we acknowledge this limitation, our conclusions regarding the relative functional roles of native and non‐native species remain robust.

## 5 Conclusion

Our study reveals that the dietary composition of frugivorous birds in Gamboa, Panama is characterised by high diversity and ecological flexibility, as evidenced by the broad range of native and non‐native plant species identified in their diets. This dietary adaptability demonstrates how these birds adjust their foraging strategies in human‐modified landscapes, mirroring broader trends observed in avian communities worldwide where many species are adjusting their foraging strategies to exploit opportunities presented by human‐altered landscapes and capitalise on novel food resources. However, our findings indicate that while non‐native plants provide alternative food resources, their integration into frugivory networks produces complex effects on network *robustness*. Critically, the inclusion of non‐native species decreased network *robustness* compared to a purely native network, with non‐native plant species, particularly taller species, playing disproportionately influential roles by reducing network *nestedness*.

Non‐native species may provide a temporary buffer against ecological disruptions by offering alternative food resources, yet they also pose risks by diverting seed dispersers away from native plants and altering long‐term community dynamics. While non‐native species may contribute to network stability by facilitating novel interactions, they may also undermine native plant recruitment and ecosystem integrity. Our findings raise particular concern about *Syzygium* species, which demonstrate high network integration as connector species while simultaneously showing significant invasive potential through their documented spread across Panama and invasiveness in other global regions. This combination of network importance and invasive capacity warrants targeted monitoring and management consideration. Future work should focus on the mechanisms by which non‐native plant characteristics influence interaction patterns and the broader ecological consequences of these shifts in the face of continued environmental change. Importantly, human‐dominated habitats like Gamboa represent increasingly common features across tropical landscapes and may serve critical functions for local biodiversity by providing essential fruit resources for frugivorous native animals.

## Author Contributions

**Adam Fell:** conceptualization (equal), formal analysis (lead), investigation (lead), methodology (lead), project administration (lead), software (lead), supervision (equal), validation (equal), visualization (lead), writing – original draft (lead), writing – review and editing (lead). **Carolina Bello:** conceptualization (equal), formal analysis (equal), methodology (equal), software (equal), supervision (equal), validation (equal), visualization (equal), writing – original draft (equal), writing – review and editing (equal). **A. Bradley Duthie:** conceptualization (supporting), formal analysis (supporting), supervision (equal), writing – original draft (supporting), writing – review and editing (supporting). **Marta Vargas:** investigation (supporting), resources (lead), validation (equal). **Alastair Skeffington:** formal analysis (supporting), software (supporting), validation (supporting). **Kristin Saltonstall:** conceptualization (supporting), formal analysis (supporting), investigation (supporting), methodology (supporting), resources (equal), software (supporting), supervision (supporting), validation (equal), visualization (supporting), writing – original draft (equal), writing – review and editing (equal). **Daisy H. Dent:** conceptualization (equal), formal analysis (equal), funding acquisition (lead), investigation (equal), methodology (equal), project administration (equal), resources (equal), software (supporting), supervision (lead), visualization (equal), writing – original draft (equal), writing – review and editing (equal).

## Funding

This work was supported by the Natural Environment Research Council (NE/s007431/1).

## Conflicts of Interest

The authors declare no conflicts of interest.

## Supporting information


**Appendix S1:** ece372620‐sup‐0001‐AppendixS1.docx.

## Data Availability

Raw information of all faecal samples is provided in Table S1, and bird species sampled with associated plant species are provided in Table S2. All model outputs are available in the main document in Tables S3 and S4.
