# Supplementary material for: Non‐Native Plants Alter Bird‐Plant Frugivory Network Structure in a Human‐Modified Tropical Landscape
Source: Ecol Evol. 2025 Dec 9;15(12):e72620. doi: 10.1002/ece3.72620 (PMC12686967; doi:10.1002/ece3.72620)
Supplement: Supplementary file 1 — Appendix S1: ece372620‐sup‐0001‐AppendixS1.docx. [file ECE3-15-e72620-s001.docx]

Supplementary material:

Supplemental Table 1. Sampling information for faecal samples collected from 93 individuals across 21 bird species in Gamboa, Central Panama between January-March 2022. Samples were collected from six different netting sites (*See Figure 1b for the locations of each site*).

| *Latin name* | | Species | | | | Date | Time | Site |
| --- | --- | --- | --- | --- | --- | --- | --- | --- |
| *Turdus grayi* | | Clay-coloured thrush | | | | 13/01/22 | 10:10 | 1 |
| *Turdus grayi* | | Clay-coloured thrush | | | | 13/01/22 | 13:20 | 1 |
| *Turdus grayi* | | Clay-coloured thrush | | | | 13/01/22 | 14:25 | 1 |
| *Turdus grayi* | | Clay-coloured thrush | | | | 13/01/22 | 15:10 | 1 |
| *Turdus grayi* | | Clay-coloured thrush | | | | 13/01/22 | 16:00 | 1 |
| *Turdus grayi* | | Clay-coloured thrush | | | | 13/01/22 | 16:25 | 1 |
| *Turdus grayi* | | Clay-coloured thrush | | | | 13/01/22 | 16:30 | 1 |
| *Turdus grayi* | | Clay-coloured thrush | | | | 13/01/22 | 16:40 | 1 |
| *Turdus grayi* | | Clay-coloured thrush | | | | 13/01/22 | 16:40 | 1 |
| *Turdus grayi* | | Clay-coloured thrush | | | | 13/01/22 | 16:45 | 1 |
| *Thraupis palmarum* | | Palm tanager | | | | 13/01/22 | 16:50 | 1 |
| *Thraupis palmarum* | | Palm tanager | | | | 13/01/22 | 17:30 | 1 |
| *Thraupis palmarum* | | Palm tanager | | | | 13/01/22 | 17:30 | 1 |
| *Turdus grayi* | | Clay-coloured thrush | | | | 14/01/22 | 09:50 | 1 |
| *Turdus grayi* | | Clay-coloured thrush | | | | 15/01/22 | 10:55 | 2 |
| *Thraupis palmarum* | | Palm tanager | | | | 15/01/22 | 11:25 | 2 |
| *Thraupis palmarum* | | Palm tanager | | | | 15/01/22 | 11:30 | 2 |
| *Thraupis palmarum* | | Palm tanager | | | | 15/01/22 | 11:35 | 2 |
| *Trogon massena* | | Slaty-tailed trogon | | | | 15/01/22 | 11:50 | 2 |
| *Trogon massena* | | Slaty-tailed trogon | | | | 15/01/22 | 11:55 | 2 |
| *Ortalis cinereiceps* | | Gray-headed chachalaca | | | | 14/01/22 | 08:00 | 1 |
| *Turdus grayi* | | Clay-coloured thrush | | | | 15/01/22 | 12:00 | 2 |
| *Thraupis palmarum* | | Palm tanager | | | | 15/01/22 | 12:10 | 2 |
| *Turdus grayi* | | Clay-coloured thrush | | | | 15/01/22 | 13:00 | 2 |
| *Thraupis palmarum* | | Palm tanager | | | | 15/01/22 | 13:20 | 2 |
| *Tityra semifasciata* | | Masked tityra | | | | 15/01/22 | 13:50 | 2 |
| *Tityra semifasciata* | | Masked tityra | | | | 15/01/22 | 15:20 | 2 |
| *Turdus grayi* | | Clay-coloured thrush | | | | 15/01/22 | 16:15 | 2 |
| *Turdus grayi* | | Clay-coloured thrush | | | | 15/01/22 | 16:45 | 2 |
| *Turdus grayi* | | Clay-coloured thrush | | | | 15/01/22 | 16:55 | 2 |
| *Ortalis cinereiceps* | | Gray-headed chachalaca | | | | 14/01/22 | 08:00 | 1 |
| *Ortalis cinereiceps* | | Gray-headed chachalaca | | | | 14/01/22 | 09:10 | 1 |
| *Tityra semifasciata* | | Masked tityra | | | | 15/01/22 | 16:55 | 2 |
| *Tityra semifasciata* | | Masked tityra | | | | 16/01/22 | 07:10 | 2 |
| *Turdus grayi* | | Clay-coloured thrush | | | | 16/01/22 | 09:45 | 2 |
| *Pitangus sulphuratus* | | Great kiskadee | | | | 16/01/22 | 10:45 | 2 |
| *Ramphastos sulfuratus* | | Keel-billed toucan | | | | 15/01/22 | 16:05 | 2 |
| *Trogon massena* | | Slaty-tailed trogon | | | | 16/01/22 | 10:55 | 2 |
| *Pitangus sulphuratus* | | Great kiskadee | | | | 16/01/22 | 14:10 | 2 |
| *Turdus grayi* | | Clay-coloured thrush | | | | 16/01/22 | 14:30 | 2 |
| *Trogon massena* | | Slaty-tailed trogon | | | | 16/01/22 | 15:00 | 2 |
| *Turdus grayi* | | Clay-coloured thrush | | | | 16/01/22 | 15:15 | 2 |
| *Thraupis episcopus* | | Blue-grey tanager | | | | 16/01/22 | 16:50 | 2 |
| *Tityra semifasciata* | | Masked tityra | | | | 16/01/22 | 17:00 | 2 |
| *Tityra semifasciata* | | Masked tityra | | | | 16/01/22 | 17:25 | 2 |
| *Turdus grayi* | | Clay-coloured thrush | | | | 17/01/22 | 08:15 | 2 |
| *Manacus vitellinus* | | Golden-collared manakin | | | | 17/01/22 | 08:25 | 2 |
| *Turdus grayi* | | Clay-coloured thrush | | | | 17/01/22 | 09:25 | 2 |
| *Turdus grayi* | | Clay-coloured thrush | | | | 17/01/22 | 09:45 | 2 |
| *Turdus grayi* | | Clay-coloured thrush | | | | 17/01/22 | 10:45 | 2 |
| *Turdus grayi* | | Clay-coloured thrush | | | | 17/01/22 | 10:45 | 2 |
| *Thraupis episcopus* | | Blue-grey tanager | | | | 17/01/22 | 11:10 | 2 |
| *Turdus grayi* | | Clay-coloured thrush | | | | 17/01/22 | 13:00 | 2 |
| *Turdus grayi* | | Clay-coloured thrush | | | | 17/01/22 | 14:00 | 2 |
| *Trogon massena* | | Slaty-tailed trogon | | | | 17/01/22 | 16:00 | 2 |
| *Manacus vitellinus* | | Golden-collared manakin | | | | 18/01/22 | 08:10 | 2 |
| *Ramphocelus dimidiatus* | | Crimson-backed tanager | | | | 18/01/22 | 10:00 | 2 |
| *Turdus grayi* | | Clay-coloured thrush | | | | 18/01/22 | 10:30 | 2 |
| *Turdus grayi* | | Clay-coloured thrush | | | | 19/01/22 | 07:45 | 2 |
| *Turdus grayi* | | Clay-coloured thrush | | | | 19/01/22 | 14:30 | 2 |
| *Trogon massena* | | Slaty-tailed trogon | | | | 19/01/22 | 10:45 | 2 |
| *Turdus grayi* | | Clay-coloured thrush | | | | 20/01/22 | 07:20 | 2 |
| *Turdus grayi* | | Clay-coloured thrush | | | | 20/01/22 | 07:45 | 2 |
| *Phylloscartes flavovirens* | | Yellow-green tyrannulet | | | | 20/01/22 | 09:00 | 2 |
| *Tityra semifasciata* | | Masked tityra | | | | 20/01/22 | 09:20 | 2 |
| *Tityra semifasciata* | | Masked tityra | | | | 20/01/22 | 09:30 | 2 |
| *Turdus grayi* | | Clay-coloured thrush | | | | 20/01/22 | 09:30 | 2 |
| *Melanerpes pucherani* | | Black-cheeked woodpecker | | | | 20/01/22 | 11:00 | 2 |
| *Melanerpes pucherani* | | Black-cheeked woodpecker | | | | 20/01/22 | 11:00 | 2 |
| *Stilpnia larvata* | | Golden-hooded tanager | | | | 20/01/22 | 11:15 | 2 |
| *Turdus grayi* | | Clay-coloured thrush | | | | 20/01/22 | 11:45 | 2 |
| *Myiodynastes maculatus* | | Streaked flycatcher | | | | 20/01/22 | 12:30 | 2 |
| *Trogon massena* | | Slaty-tailed trogon | | | | 21/01/22 | 07:30 | 2 |
| *Turdus grayi* | | Clay-coloured thrush | | | | 21/01/22 | 08:30 | 2 |
| *Turdus grayi* | | Clay-coloured thrush | | | | 23/01/22 | 08:45 | 2 |
| *Turdus grayi* | | Clay-coloured thrush | | | | 23/01/22 | 09:15 | 2 |
| *Turdus grayi* | | Clay-coloured thrush | | | | 23/01/22 | 10:45 | 2 |
| *Melanerpes pucherani* | | Black-cheeked woodpecker | | | | 23/01/22 | 14:15 | 2 |
| *Trogon caligatus* | | Gartered trogon | | | | 23/01/22 | 15:00 | 2 |
| *Columbina talpacoti* | | Ruddy ground dove | | | | 26/01/22 | 15:45 | 3 |
| *Momotus subrufescens* | | Whooping motmot | | | | 27/01/22 | 08:00 | 3 |
| *Trogon caligatus* | | Gartered trogon | | | | 27/01/22 | 14:30 | 3 |
| *Ortalis cinereiceps* | | Gray-headed chachalaca | | | | 28/01/22 | 09:00 | 3 |
| *Ortalis cinereiceps* | | Gray-headed chachalaca | | | | 30/01/22 | 09:45 | 3 |
| *Trogon massena* | | Slaty-tailed trogon | | | | 30/01/22 | 10:00 | 2 |
| *Ortalis cinereiceps* | | Gray-headed chachalaca | | | | 14/02/22 | 09:20 | 1 |
| *Leptotila verreauxi* | | White-tipped dove | | | | 15/02/22 | 10:55 | 1 |
| *Momotus subrufescens* | | Whooping motmot | | | | 15/02/22 | 11:45 | 1 |
| *Ramphastos sulfuratus** | | Keel-billed toucan | | | | 22/02/22 | 11:00 | 4 |
| *Trogon massena* | | Slaty-tailed trogon | | | | 24/02/22 | 16:30 | 4 |
| *Mimus gilvus* | | Tropical mockingbird | | | | 12/03/22 | 07:45 | 5 |
| *Patagioenas cayennensis* | | Pale vented pigeon | | | | 15/03/22 | 06:30 | 6 |
| *Brotogeris jugularis* | | Orange-chinned parakeet | | | | 16/03/22 | 07:45 | 6 |
| ** Collected two samples from this individual* | | | | | |  |  |  |
| Site coordinates: | |  | | | |  |  |  |
| 1 | 9.1176 N, -79.6995 W | |  | **4** | 9.1214 N, -79.6942 W | | | |
| 2 | 9.1271 N, -79.6946 W | |  | **5** | 9.1180 N, -79.6969 W | | | |
| 3 | 9.1192 N, -79.6981 W | |  | **6** | 9.1187 N, -79.6958 W | | | |

Supplementary Table 2. Bird species sampled in Central Panama (n = 21) and the total number of individual samples per species (total samples = 94 ). Data presented includes the number of unique plant species identified in the faeces of each bird species, genera and families found across all samples of the same bird species and the average proportion of native, non-native, fleshy-fruiting, non-fleshy-fruiting, woody stemmed, and non-woody stemmed plant species found in each bird species.

| **Bird species** | **Number of**  **samples** | **Unique plant**  **species** | **Unique plant**  **genera** | **Unique plant**  **families** | **Native**  **species (%)** | **Non-native**  **species (%)** | **Fleshy fruit**  **species (%)** | **Non-fleshy fruit**  **species (%)** | **Woody**  **species (%)** | **Non-woody**  **species (%)** |
| --- | --- | --- | --- | --- | --- | --- | --- | --- | --- | --- |
| *Brotogeris jugularis* | 1 | 9 | 8 | 7 | 100 | 0 | 33 | 67 | 100 | 0 |
| *Columbina talpacoti* | 1 | 5 | 5 | 5 | 40 | 60 | 40 | 60 | 60 | 40 |
| *Leptotila verreauxi* | 1 | 4 | 5 | 4 | 80 | 20 | 40 | 60 | 40 | 60 |
| *Manacus vitellinus* | 2 | 9 | 7 | 5 | 78 | 22 | 78 | 22 | 100 | 0 |
| *Melanerpes pucherani* | 3 | 16 | 15 | 12 | 81 | 19 | 50 | 50 | 88 | 12 |
| *Mimus gilvus* | 1 | 13 | 10 | 9 | 92 | 8 | 54 | 46 | 100 | 0 |
| *Momotus subrufescens* | 2 | 6 | 5 | 4 | 67 | 33 | 83 | 17 | 100 | 0 |
| *Myiodynastes maculatus* | 1 | 9 | 8 | 6 | 78 | 22 | 56 | 44 | 89 | 11 |
| *Ortalis cinereiceps* | 6 | 21 | 18 | 13 | 76 | 24 | 62 | 38 | 76 | 24 |
| *Patagioenas cayennensis* | 1 | 3 | 2 | 2 | 67 | 33 | 100 | 0 | 100 | 0 |
| *Pitangus sulphuratus* | 2 | 9 | 7 | 6 | 56 | 44 | 78 | 22 | 78 | 22 |
| *Phylloscartes flavovirens* | 1 | 3 | 3 | 3 | 100 | 0 | 100 | 0 | 100 | 0 |
| *Ramphastos sulfuratus* | 3 | 22 | 17 | 15 | 91 | 9 | 68 | 32 | 95 | 5 |
| *Ramphocelus dimidiatus* | 1 | 9 | 9 | 7 | 100 | 0 | 33 | 67 | 67 | 33 |
| *Stilpnia larvata* | 1 | 7 | 6 | 4 | 86 | 14 | 86 | 14 | 100 | 0 |
| *Thraupis episcopus* | 2 | 13 | 11 | 9 | 77 | 23 | 62 | 38 | 85 | 15 |
| *Thraupis palmarum* | 8 | 24 | 20 | 16 | 79 | 21 | 75 | 25 | 83 | 17 |
| *Tityra semifasciata* | 8 | 22 | 21 | 15 | 68 | 32 | 77 | 23 | 82 | 18 |
| *Trogon caligatus* | 2 | 17 | 16 | 14 | 76 | 24 | 65 | 35 | 76 | 24 |
| *Trogon massena* | 9 | 42 | 38 | 20 | 86 | 14 | 55 | 45 | 81 | 19 |
| *Turdus grayi* | 38 | 71 | 55 | 33 | 73 | 27 | 62 | 38 | 80 | 20 |

Supplementary Table 3 – Effects of native status, plant height, seed mass, degree (the number of interactions per species) and their interactions on plant species persistence in the frugivory network, as shown in the negative binomial regression model. Estimates represent the effect size of each predictor, with positive values indicating increased persistence. All continuous variables were centred and scaled prior to fitting the model. Significant effects are indicated with (*), and marginal effects are indicated with (.).

|  | **Estimate** | **Std. Error** | **z value** | **Pr(>\|z\|)** |
| --- | --- | --- | --- | --- |
| Intercept | 5.554 | 0.202 | 27.526 | < 0.001 *** |
| Native (Yes) | 0.466 | 0.215 | 2.173 | 0.029 * |
| Plant height | -0.004 | 0.152 | -0.025 | 0.980 |
| Seed mass | -0.588 | 0.367 | -1.604 | 0.109 |
| Degree | 0.393 | 0.069 | 5.657 | < 0.001 *** |
| Native (Yes) * Plant height | 0.640 | 0.376 | 1.705 | 0.088 . |
| Native (Yes) * Seed weight | -0.110 | 0.166 | -0.665 | 0.506 |

Supplementary Table 4 – Effects of nativeness, plant height, seed mass, degree and their interactions on the total number of secondary extinctions caused by plant removals across all replications, as shown in the negative binomial regression model. Estimates represent the effect size of each predictor, with positive values indicating increased persistence. All continuous variables were centred and scaled prior to fitting the model. Significant effects are indicated with (*), and marginal effects are indicated with (.).

|  | **Estimate** | **Std. Error** | **z value** | **Pr(>\|z\|)** |
| --- | --- | --- | --- | --- |
| Intercept | 5.732 | 0.146 | 39.33 | < 0.001 *** |
| Native (Yes) | -0.414 | 0.146 | -2.83 | 0.005 ** |
| Plant height | 0.405 | 0.209 | 1.93 | 0.053. |
| Seed mass | 0.009 | 0.054 | 0.16 | 0.874 |
| Degree | -1.046 | 0.161 | -6.49 | < 0.001 *** |
| Native (Yes) * Plant height | -0.438 | 0.223 | -1.97 | 0.049 * |


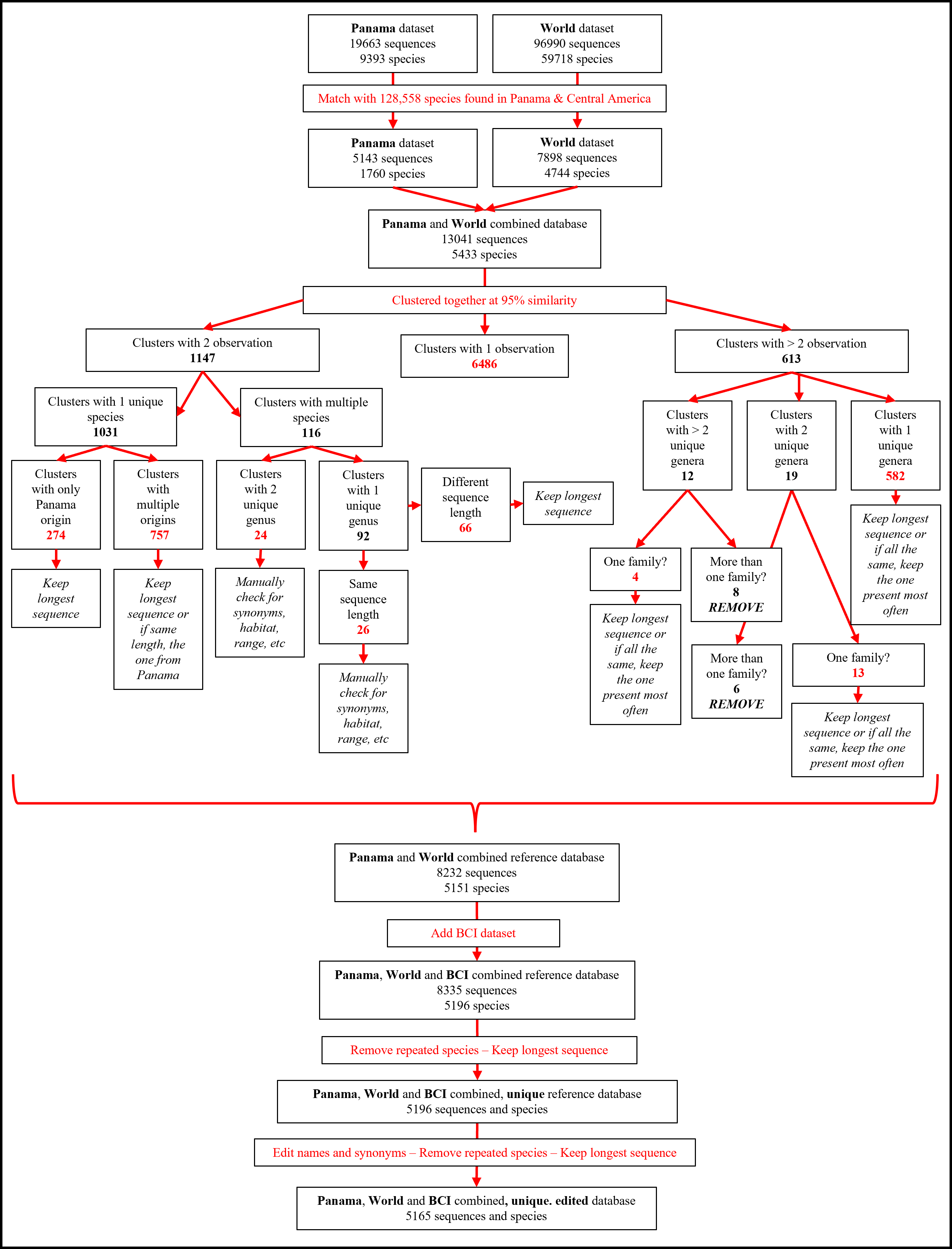


Supplementary Figure 1. Flow diagram illustrating the decisions made to create our customised ITS2 plant reference database for species found in Panama and Central America. The final database included 5165 unique species. Clusters are groups of sequences that matched at a 95% level and were grouped together as the same species. Decisions were made until there was more than one family in a group, at which point all observations within that group were discarded. Red text indicates actions taken and red numbers indicate the total sequences kept from each cluster.


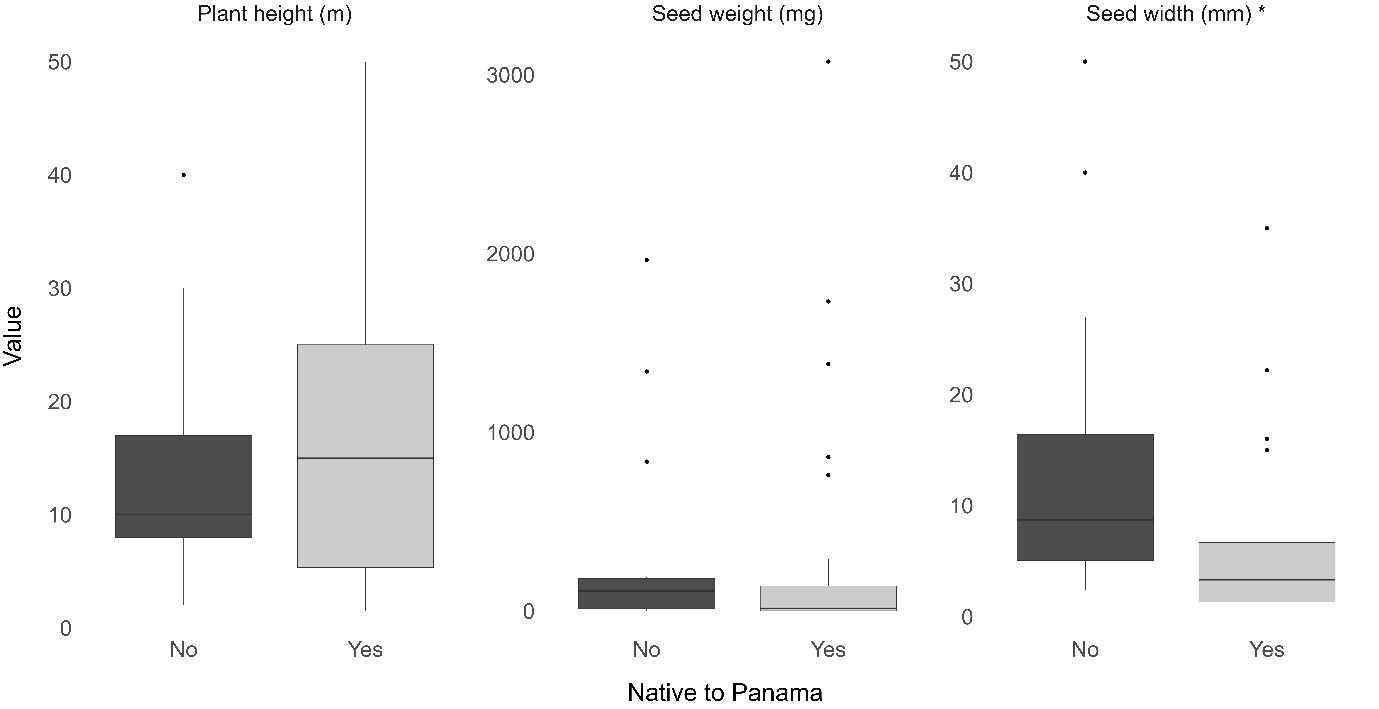


Supplementary Figure 2. Boxplots showing the comparison of traits between native and non-native plant species that were consumed by frugivorous birds. Traits include average plant height, seed width and seed mass, as presented by the NeoFrugivory database (Fuzessy & Pizo, 2025), the Botanical Information and Ecology Network (BIEN) database (Maitner et al, 2017), the TRY Plant Trait Database (Kattge et al, 2011), and the SER Seed Information Database (SID, 2023).


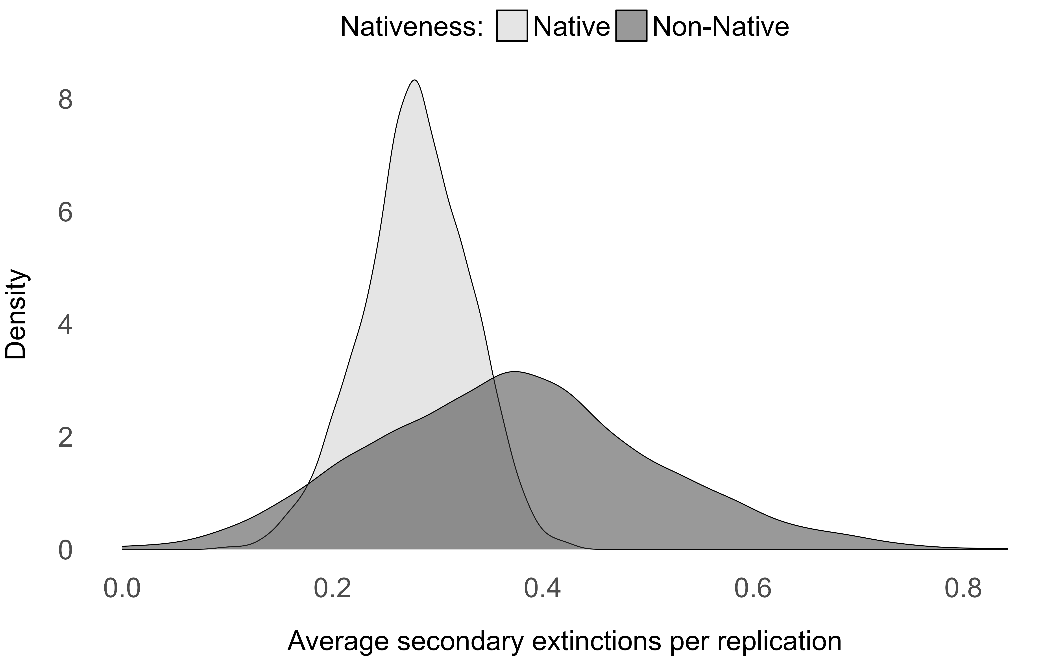


Supplementary Figure 3**.** Density curves illustrating the distribution of the average number of secondary extinctions caused by native and non-native plant species removals in every extinction replication.


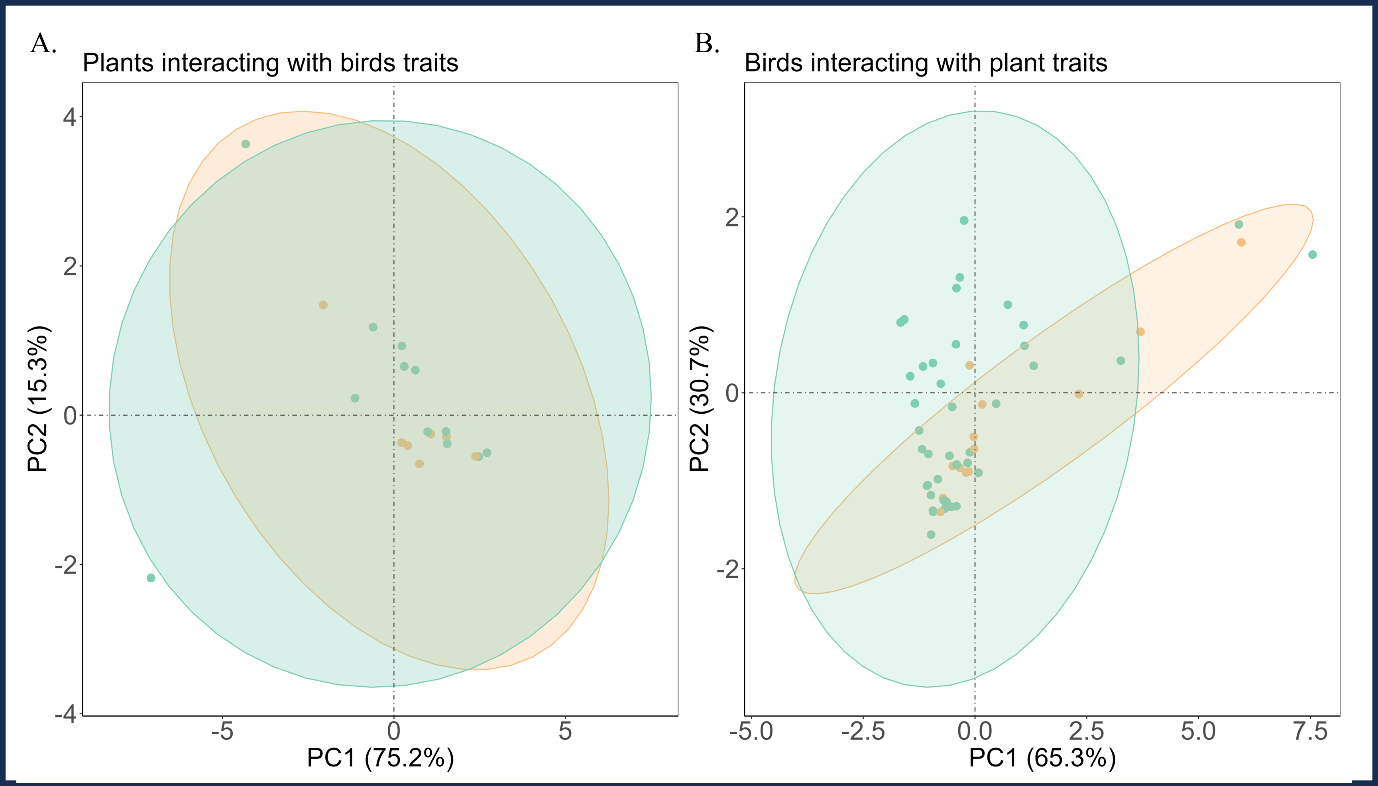


Supplementary Figure 4. Principal component analysis (PCA) of plant species distributions weighted by interaction frequency (FOO_per_bird_species/100) in (A) bird trait space and (B) plant trait space. Green ellipses represent native plants; orange ellipses represent non-native plants. In the bird trait space analysis (A), plant species are ordinated based on the morphological traits of their avian dispersers (*average weight, beak width, beak length, beak depth, tarsus length, wing length, secondary length, and tail length*). In the plant trait space analysis (B), plant species are ordinated based on their own morphological characteristics (*seed width, plant height, and seed weight*). Confidence ellipses represent 95% confidence intervals. Percentage values on axes indicate the proportion of variance explained by each principal component.
